# Security of Quantum Key Distribution with One-Time-Pad-Protected Error Correction and Its Performance Benefits

**DOI:** 10.3390/e27101032

**Published:** 2025-10-01

**Authors:** Roman Novak

**Affiliations:** Department of Communication Systems, Jožef Stefan Institute, 1000 Ljubljana, Slovenia; roman.novak@ijs.si

**Keywords:** quantum key distribution, quantum information, error correction, unconditional security, information reconciliation, security proof, one-time pad encryption

## Abstract

In quantum key distribution (QKD), public discussion over the authenticated classical channel inevitably leaks information about the raw key to a potential adversary, which must later be mitigated by privacy amplification. To limit this leakage, a one-time pad (OTP) has been proposed to protect message exchanges in various settings. Building on the security proof of Tomamichel and Leverrier, which is based on a non-asymptotic framework and considers the effects of finite resources, we extend the analysis to the OTP-protected scheme. We show that when the OTP key is drawn from the entropy pool of the same QKD session, the achievable quantum key rate is identical to that of the reference protocol with unprotected error-correction exchange. This equivalence holds for a fixed security level, defined via the diamond distance between the real and ideal protocols modeled as completely positive trace-preserving maps. At the same time, the proposed approach reduces the computational requirements: for non-interactive low-density parity-check codes, the encoding problem size is reduced by the square of the syndrome length, while privacy amplification requires less compression. The technique preserves security, avoids the use of QKD keys between sessions, and has the potential to improve performance.

## 1. Introduction

A comprehensive, self-contained proof of security for quantum key distribution (QKD) was presented in [1] that considers the effects of finite resources. The analysis includes both entanglement-based and prepare-and-measure protocols within a unified framework, using a security reduction to relate the latter to the former. Specifically, the considered protocols correspond to variants of BBM92 [2] and BB84 [3], respectively.

When a QKD protocol is represented as a completely positive trace-preserving (CPTP) map, its security can be quantified by its operational distinguishability from an ideal protocol, which is defined as one in which the final keys are independent, uniformly distributed random strings. A QKD protocol that is Δ-secure has a maximum distinguishing probability of $\frac{1}{2}\left(1+\Delta \right)$ in an optimal experiment. Formally, Δ is the diamond distance between the actual and ideal CPTP maps and extends the notion of trace distance from quantum states to quantum channels.

A critical stage in QKD is key reconciliation, where error-correction information is exchanged over a public channel. This stage can significantly influence the security. To prevent the leakage of information to a potential adversary, encryption of reconciliation data has been proposed. Among the possible schemes, the one-time pad cipher (OTP) is of particular interest due to its unconditional security, which remains intact even against quantum adversaries, provided that the strict requirements are met. The main limitation of the OTP lies in its demand for a secret key whose length is at least equal to the length of the message and which must be securely exchanged in advance between the communicating parties. For instance, in [4], a standalone non-quantum key distribution method based on optical noise and supplemented by privacy amplification is proposed to address this requirement.

The explicit integration of the OTP scheme into QKD has been explored in several studies. In [5], the OTP is used to encrypt error-correction data in order to decouple error correction from privacy amplification. In this approach, Alice and Bob must initially share an OTP key whose length is equal to the requirements of a full QKD session, and the cost of this initial key is to be offset by generating a longer quantum key. A similar strategy is adopted in [6], where part of the QKD key generated in a previous session is reused as the OTP key for the following session. While both methods are effective in principle, they require an initial pre-shared key. Consequently, a complete proof of security should consider both the initialization phase and the security implications of the key-chaining process.

In this paper, we present an alternative use of the OTP cipher within QKD that avoids pre-shared keys and chaining. Specifically, a designated block of the raw key obtained within the same QKD session is used as the OTP key to encrypt the error-correction data for the remaining portion of the raw key. A key distinction from earlier proposals is that the OTP keys used by Alice and Bob are necessarily different. Since the OTP keys originate from the same QKD session in which they are applied, eliminating the need for key reuse across sessions, the protocol allows a formal assessment of security on a per-session basis alone.

We extend the non-asymptotic security proof of Tomamichel and Leverrier [1], which analyzes the security of QKD at finite key lengths while allowing for a small probability of failure. In this setting, we show that the OTP extension achieves the same quantum key rate as the conventional protocol—where the key rate is defined as the ratio of the final key length to the total number of quantum systems shared between Alice and Bob—at the desired security level. No additional assumptions are required for the OTP keys beyond those that already apply to the raw key, which are briefly summarized in the next section and described in detail in [1] (pp. 7–9).

While the proposed OTP extension of the QKD protocol does not change its security level, it provides a practical advantage by reducing the computational requirements of error correction. We illustrate these benefits using low-density parity-check (LDPC) codes, a subclass of forward error-correction (FEC) codes that allow the receiver to detect and correct errors without retransmission. We show that the size of the encoding problem decreases by the square of the syndrome length, while the size of the decoding problem remains the same. Moreover, the QKD session requires less compression during privacy amplification.

We begin with an overview of the QKD reference protocol and summarize the main conclusions of the original security proof. The formalism and notation introduced in [1] (pp. 3–7) are adopted, and readers are encouraged to consult that work for a full treatment of the proof. A complete restatement of all theorems and lemmas is not necessary here. However, we highlight those assumptions that are relevant to the modeling of the OTP extension and the security proof.

The structure of the paper is as follows. Section 2 recalls the entanglement-based QKD protocol and introduces the notation used in the analysis. Section 3 presents the modification of the error-correction step, including a visualization of the classical and quantum systems involved. Section 4 establishes theoretical bounds on the length of the error-correction data for both noisy and noise-free channels. In Section 5, we adjust the mathematical model of the QKD reference protocol, which serves as the basis for extending the original security proof to the OTP-enhanced protocol in Section 6. Section 7 evaluates the performance benefits of the modified error-correction scheme when implemented with LDPC codes. Finally, Section 8 concludes the paper.

## 2. Reference Protocol

The security proof in [1] applies to a variant of the entanglement-based QKD protocol introduced in [2] and is subsequently extended to prepare-and-measure schemes with essentially identical results. For completeness, we briefly recall the relevant elements of this otherwise well-known protocol.

The protocol takes as input a bipartite quantum state $\rho_{AB}$ and outputs two, typically identical, binary strings $K_{A}$ and $K_{B}$ representing the final keys held by Alice and Bob, respectively. The protocol may also abort under one of two conditions: failure of parameter estimation, indicated by the flag $F^{\mathrm{pe}}$, or failure of error correction, indicated by the flag $F^{\mathrm{ec}}$.

Alice and Bob each start with *m* quantum systems, where the specific physical mechanism by which they are obtained is left unspecified. These systems are modeled as tensor products of local Hilbert spaces of the systems *A* and *B*. The proof in [1] relies on several assumptions—such as deterministic detection, commuting measurements, and measurement complementarity—which, while necessary for the original proof, are not repeated here. Likewise, the sifting procedure used to ensure basis matching has been completed in advance.

At the beginning of the protocol, Alice generates a set of random seeds and transmits them to Bob via the authenticated classical communication channel. These seeds determine the random selection of a subset of raw key bits for parameter estimation ($S^{\Pi }$), the choice of measurement bases for parameter estimation ($S^{\Xi }$), and the measurement bases of the remaining systems used in key extraction ($S^{\Theta }$). Two other seeds, $S^{H_{\mathrm{ec}}}$ and $S^{H_{\mathrm{pa}}}$, are used to randomly select particular hash functions from a universal family of hash functions. For clarity, we explicitly introduce these seeds when they appear in the following analysis.

The measurement outcomes of the *m* quantum systems—on both Alice’s and Bob’s sides—are recorded in binary registers. Each register is partitioned into two disjoint segments according to a random selection procedure controlled by the shared seeds: a segment of length *k* reserved for parameter estimation, denoted by *V* on Alice’s side and *W* on Bob’s side, and a segment of length *n* reserved for key distillation, denoted by *X* on Alice’s side and *Y* on Bob’s side.

In the parameter estimation step, Alice transmits a transcript $C^{V}$ of her *V* over the public channel. After receiving $C^{V}$, Bob compares *V* with his corresponding *W* and determines whether the observed error rate is below a predefined threshold δ. If the threshold is exceeded, Bob sets $F^{\mathrm{pe}}=\emptyset$, and the protocol aborts.

The error-correction procedure is characterized by the quintuple $\left\{t,r,\mathrm{synd},\mathrm{corr},H_{\mathrm{ec}}\right\}$, where the details of syndrome-based error correction are explained in Section 7. In short, to reconcile discrepancies between *X* and *Y*, Alice computes an error-correction syndrome Z=synd(X) and transmits its public transcript $C^{Z}$ of length *r* over the authenticated classical channel. Bob applies an efficient correction algorithm $\hat{X}=\mathrm{corr}\left(Y,Z\right)$, producing $\hat{X}$ as his best estimate of Alice’s *X*.

Both parties then check the success of the error correction by computing the hash values of *X* and $\hat{X}$, respectively, using a randomly chosen function from a family of universal hash functions $H_{\mathrm{ec}}$. The choice of hash function is determined by the seed $S^{H_{\mathrm{ec}}}$ generated by Alice and transmitted over the public channel. After receiving Alice’s hash transcript $C^{T}$ of length *t*, Bob compares the results and sets the error-correction flag $F^{\mathrm{ec}}$ accordingly; if $F^{\mathrm{ec}}=\emptyset$, the protocol aborts.

In the final stage, privacy amplification is performed to meet the prescribed security parameters. A random hash function $H_{\mathrm{pa}}$ from a family of universal hash functions $H_{\mathrm{pa}}$ is selected using the seed $S^{H_{\mathrm{pa}}}$, generated and publicly announced by Alice. The final keys, of length *l*, are computed as $K_{A}=H_{\mathrm{pa}}\left(X\right)$ and $K_{B}=H_{\mathrm{pa}}\left(\hat{X}\right)$.

We summarize the notation used in the modeling and proof in Table 1, similar to [1], with additional registers introduced. The notation used to describe error-correction algorithms is given separately in Section 7.

## 3. OTP-Protected Error Correction

We make no restrictions on the choice of error-correction method, except that it must operate in a non-interactive, or one-way, mode. In [1], the authors propose the use of a linear code defined by a parity-check matrix. While interactive error-correction methods could, in principle, also be protected by OTP encryption, such an approach falls outside the scope of our current analysis.

**Figure 1:**
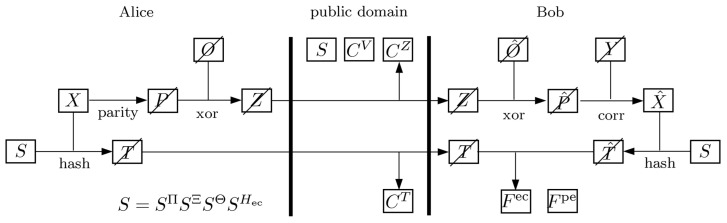
State of the classical and quantum systems during and after error correction with the one-time pad (OTP) protection.

To describe the extension of the QKD protocol, we adopt the representation in Figure 1, which provides a modified view of the joint evolution of the classical and quantum systems during and after error correction, based on the original representation in [1]. In this diagram, the boxes represent subsystems accessible to Alice, Bob, and the public channel, while temporary classical systems are indicated by crossed-out boxes. The preceding and following steps of the protocol are identical to those in the reference formulation.

In the proposed extension, Alice and Bob agree on a subset of their raw quantum key, which serves as a one-time pad. The quantum representations of the corresponding classical OTP registers, denoted by *O* on Alice’s side and $\hat{O}$ on Bob’s side, contain the respective measurement outcomes of their initial quantum systems *A* and *B*. The formal introduction of these newly defined registers, together with the measurement maps that determine their contents, can be found in Section 5.

The modified procedure is as follows. Alice first computes the error-correction data P=parity(X) and then applies the OTP protection by forming Z=xor(P,O), where xor denotes the bitwise addition modulo 2. The public transcript $C^{Z}$ of register *Z* is then transmitted via the authenticated classical channel.

After obtaining $C^{Z}$, Bob reverses the transformation by calculating $\hat{P}=\mathrm{xor}\left(Z,\hat{O}\right)$. He then applies the error-correction algorithm to obtain $\hat{X}=\mathrm{corr}\left(Y,\hat{P}\right)$. The estimate $\hat{X}$ is then processed analogously to the reference protocol: the two parties compute the hash values *T* and $\hat{T}$, respectively, with Bob performing the comparison of the values to verify successful reconciliation, setting the error-correction flag $F^{\mathrm{ec}}$ accordingly. Depending on the result, the protocol either aborts or proceeds with privacy amplification, as described in Section 2.

## 4. Syndrome and Parity Lengths

For an *n*-bit raw key transmitted over a binary symmetric channel (BSC) with a crossover probability *p*, the minimum syndrome length *r* required for error correction is determined by the entropy of the error pattern, namely, nh(p), where h(p) denotes the binary entropy, $h\left(p\right)=-p\log_{2}p-\left(1-p\right)\log_{2}\left(1-p\right)$. This bound follows directly from Shannon’s source coding theorem [7] and the Slepian–Wolf theorem [8] for lossless source coding with side information, and was formalized by Brassard and Salvail in [9] for syndrome-based error correction over an error-free channel.

In practice, error-correction codes are not theoretically optimal. Their efficiency is commonly quantified by a parameter $f_{e}>1$, with larger $f_{e}$ indicating reduced efficiency. The actual number of syndrome bits required is therefore

(1) $$r=f_{e}nh\left(p\right).$$

Since the OTP scheme introduces additional errors, the problem becomes equivalent to error correction over a noisy channel, which increases the required redundancy. In this case, transmitting nh(p) correction bits would itself require correction, adding another $nh^{2}\left(p\right)$ bits, which in turn would require $nh^{3}\left(p\right)$ bits, and so forth. The minimum number of correction bits in this setting is therefore

(2) $$\sum_{i=1}^{\infty }nh^{i}\left(p\right)=\frac{nh(p)}{1-h(p)},$$

where h(p)<1, as first derived in [7] from the channel capacity constraint. Accounting for inefficiency, this expression becomes

(3) $$r_{\mathrm{noisy}}=\frac{f_{e}nh\left(p\right)}{1-f_{e}h\left(p\right)}$$

for $f_{e}h\left(p\right)<1$. Since what is exchanged in this context is not a syndrome but more general redundancy information, it is more appropriate—by analogy with classical telecommunications—to use the term parity exchange instead of syndrome exchange. Accordingly, in the OTP-protected scheme, the error-correction quintuple is updated to $\left\{t,r_{\mathrm{noisy}},\mathrm{parity},\mathrm{corr},H_{\mathrm{ec}}\right\}$.

## 5. Revised Mathematical Model

We now revise the mathematical model of the QKD protocol from [1], which will serve as the basis for extending the original security proof to the OTP-enhanced version in the following section. As mentioned above, we assume that Alice and Bob each have a collection of *m* individual quantum systems, with Alice’s systems described by the tensor product of Hilbert spaces $A=A_{1}⊗A_{2}⊗\ldots ⊗A_{m}$, and Bob’s systems analogously by $B=B_{1}⊗B_{2}⊗\ldots ⊗B_{m}$. The states of these systems are arbitrary, finite-dimensional, and otherwise unrestricted, so that the joint input state is fully described by a density operator $\rho_{AB}$.

Once the random seeds have been distributed over the authenticated public channel, the global state of the protocol is described by $\rho_{ABS^{\Pi }S^{\Xi }S^{\Theta }}$. Each of the classical registers is represented in the model as a quantum state. For example, the register encoding the seed $S^{\Pi }$ is described as the maximally mixed state

(4) ρSΠ=∑π∈Πm,k1mk|π⟩⟨π|SΠ,

where $\Pi_{m,k}$ is the set of all subsets of size *k* chosen from *m* elements, and ${\left\{|\pi \rangle \right\}}_{\pi \in \Pi_{m,k}}$ forms an orthonormal basis of the register space.

Since the OTP modification only affects the error-correction phase, the modeling of the parameter estimation remains identical to that in [1]. Measurements are represented as CPTP maps that transform quantum systems into the content of a classical register. A general measurement is defined as

(5) $$M_{A\to X}:\rho_{AB}\mapsto \sigma_{XB}=\sum_{x\in X}|x\rangle {\left\langle x\right|}_{X}⊗{\mathrm{tr}}_{A}\left\{M_{A}^{x}\rho_{AB}{\left(M_{A}^{x}\right)}^{†}\right\},$$

where *A* denotes the measured subsystem, *X* the resulting register, and $M_{A}^{x}$ the measurement operator yielding outcome *x*. Without going into the explicit representation in orthonormal bases, we denote by $\tau_{ABVWS^{\Pi }S^{\Xi }S^{\Theta }}$ the state obtained after applying the measurement map to the subsystems selected by $S^{\Pi }$ and storing the outcomes in the registers *V* on Alice’s side and *W* on Bob’s side:

(6) $$\tau_{ABVWS^{\Pi }S^{\Xi }S^{\Theta }}=M_{AB\to VW|S^{\Pi }S^{\Xi }}\left(\rho_{AB}⊗\rho_{S^{\Pi }}⊗\rho_{S^{\Xi }}⊗\rho_{S^{\Theta }}\right).$$

The measurement process is conceptually divided into two groups: (i) measurements used for parameter estimation and (ii) measurements used for extracting the secret key. This division is formal and has no impact on the practical realization of the protocol. Note that the measurement operators depend on bases determined by the random seed $S^{\Xi }$, represented as the maximally mixed classical state $\rho_{S^{\Xi }}$. In addition, the state τ in (6) is extended by a similarly constructed $\rho_{S^{\Theta }}$.

To incorporate the OTP scheme, we need to change the second group of measurements. Instead of the total measurement map defined in [1] as

(7) $$M_{AB\to VWXY|S^{\Pi }S^{\Xi }S^{\Theta }}:=M_{AB\to XY|S^{\Pi }S^{\Theta }}\circ M_{AB\to VW|S^{\Pi }S^{\Xi }},$$

we introduce the modified map

(8) $$M_{AB\to VWXOY\hat{O}|S^{\Pi }S^{\Xi }S^{\Theta }}:=M_{AB\to XOY\hat{O}|S^{\Pi }S^{\Theta }}\circ M_{AB\to VW|S^{\Pi }S^{\Xi }},$$

in which the raw keys are split into two components: *X* and *O* on Alice’s side, and *Y* and $\hat{O}$ on Bob’s side. The OTP blocks satisfy $\left|O|=\right|\hat{O}|=r_{\mathrm{noisy}}$, while the remaining raw key lengths are $\left|X\right|=\left|Y\right|=m-k-r_{\mathrm{noisy}}$. The partitioning itself is arbitrary, provided that both parties select the same subset of the raw key. After the quantum systems *A* and *B* have been discarded, the resulting classical state is

(9) $$\sigma_{VWXOY\hat{O}S^{\Pi }S^{\Xi }S^{\Theta }}={\mathrm{tr}}_{AB}\left(M_{AB\to XOY\hat{O}|S^{\Pi }S^{\Theta }}\left(\tau_{ABVWS^{\Pi }S^{\Xi }S^{\Theta }}\right)\right).$$

Since the parameter estimation phase is modeled identically to [1], Equation (9) conditioned on the parameter estimation outcome is

(10) $$\sigma_{VWXOY\hat{O}S^{\Pi }S^{\Xi }S^{\Theta }F^{\mathrm{pe}}}=E_{\mathrm{pe}}\left(\sigma_{VWXOY\hat{O}S^{\Pi }S^{\Xi }S^{\Theta }}\right),$$

where $E_{\mathrm{pe}}\left(\cdot \right)$ denotes the CPTP map corresponding to the parameter estimation function

(11) $$\mathrm{pe}\left(v,w\right):{\left\{0,1\right\}}^{k}\times {\left\{0,1\right\}}^{k}\to \left\{\emptyset ,✓\right\},$$

which determines the quantum representation of the flag $F^{\mathrm{pe}}$. Note that, for a general function f:X→Y, the corresponding CPTP map is defined as

(12) $$E_{f}\left(\cdot \right)=\sum_{x\in X}{\left|f(x)\right\rangle }_{Y}|x\rangle {\left\langle x\right|}_{X}\cdot |x\rangle {\left\langle x\right|}_{X}{\left\langle f(x)\right|}_{Y},$$

which leaves the register *X* intact while appending a new register *Y*, i.e., $E_{f}:X\to XY$.

By renaming *V* as transcript $C^{V}$ published on the public channel and discarding *W*, we obtain $\sigma_{XOY\hat{O}C^{V}S^{\Pi }S^{\Xi }S^{\Theta }F^{\mathrm{pe}}}$, which is the input state for the error correction and OTP encryption.

Let $E_{\mathrm{parity}}$, $E_{\mathrm{xor}}$, and $E_{\mathrm{corr}}$ denote the CPTP maps implementing the respective functions as given in Section 3, and let $E_{\mathrm{ec}}$ denote the map that computes the verification hash *T* and the success flag $F^{\mathrm{ec}}$. The final state after error correction is then represented by the composition

(13) $$\begin{array}{c} \sigma_{X\hat{X}C^{V}C^{Z}C^{T}S^{\Pi }S^{\Xi }S^{\Theta }S^{H_{\mathrm{ec}}}F^{\mathrm{pe}}F^{\mathrm{ec}}}\\ \;={\mathrm{tr}}_{YO\hat{O}P\hat{P}}\left\{E_{\mathrm{ec}}\circ E_{\mathrm{corr}}\circ E_{\mathrm{xor}}\circ E_{\mathrm{xor}}\circ E_{\mathrm{parity}}\left(\sigma_{XOY\hat{O}C^{V}S^{\Pi }S^{\Xi }S^{\Theta }F^{\mathrm{pe}}}⊗\rho_{S^{H_{\mathrm{ec}}}}\right)\right\} \end{array},$$

where the new subsystems $C^{Z}$, $C^{T}$, $S^{H_{\mathrm{ec}}}$, and $F^{\mathrm{ec}}$ arise from the respective CPTP maps and the inclusion of the uncorrelated quantum representation of the random seed $\rho_{S^{H_{\mathrm{ec}}}}$.

The modeling of the privacy amplification remains unchanged from [1]. Specifically, the distilled keys are compressed via a universal hash function $H_{\mathrm{pa}}\in H_{\mathrm{pa}}$, which is selected and publicly announced by Alice using a random seed $S^{H_{\mathrm{pa}}}$. The final keys are $K_{A}=H_{\mathrm{pa}}\left(X\right)$ and $K_{B}=H_{\mathrm{pa}}\left(\hat{X}\right)$, where the process is represented by a CPTP map $E_{\mathrm{pa}}$. The final state of the protocol is therefore

(14) $$\omega_{K_{A}K_{B}CSF}={\mathrm{tr}}_{X,\hat{X}}\left\{E_{\mathrm{pa}}\left(\sigma_{X\hat{X}CSF}⊗\rho_{S^{H_{\mathrm{pa}}}}\right)\right\},$$

where CSF denotes the collection of all transcripts, seeds, and flags exposed on the public channel. This state has the same structural form as in [1], although the sizes and interdependencies of the subspaces differ due to the OTP modifications.

## 6. Security Proof Extension

We quantify the distinguishability between the CPTP map representing the QKD protocol formalized above and that of an ideal protocol, in which the final keys $K_{A}$ and $K_{B}$ are replaced by independent, uniformly distributed random bit strings. For the entanglement-based formulation, this distinguishability is evaluated by the diamond distance

(15) $$\Delta =\underset{\rho_{ABE}\in S\left(ABE\right)}{\sup}||\mathrm{qkd}\left(\rho_{ABE}\right)-\mathrm{qkd\_ideal}\left(\rho_{ABE}\right){\left|\right|}_{\mathrm{tr}},$$

where the supremum is taken over all normalized states on the joint system ABE. Here, *E* denotes the purifying environment controlled by the eavesdropper, Eve. It suffices to assume |E|=|A||B|. Since purification represents the strongest possible adversary, any attack by Eve, whether collective, coherent, or memory-based, can be modeled as her holding the purification. However, it is important to emphasize that real-world security can be compromised if the underlying assumptions are violated. For example, vulnerabilities can arise if the source of the quantum state deviates from the modeled behavior or if the assumption of a sealed laboratory does not hold.

In [1], the authors establish an upper bound on Δ by uniformly bounding the trace distance of the protocol’s final state from the corresponding ideal state. More precisely, they consider

(16) $$\left|\right|\omega_{K_{A}K_{B}SCFE\wedge F=\left(✓,✓\right)}-\chi_{K_{A}K_{B}}⊗\omega_{SCFE\wedge F=(✓,✓)}{\left|\right|}_{\mathrm{tr}},$$

where the notation ∧F=(✓,✓) denotes the sub-normalized state [1] (p. 5) conditioned on both successful parameter estimation and successful error correction. The ideal key of length *l* is modeled as the maximally mixed state

(17) $$\chi_{K_{A}K_{B}}=\sum_{k\in {\left\{0,1\right\}}^{l}}\frac{1}{2^{l}}|k\rangle {\left\langle k\right|}_{K_{A}}⊗|k\rangle {\left\langle k\right|}_{K_{B}},$$

defined in the orthonormal basis ${\left\{|k\rangle \right\}}_{k\in {\left\{0,1\right\}}^{l}}$.

The derivation of Δ and its proof can be summarized as follows. Lemma 1 of [1] (p. 15) establishes that Δ can be decomposed into two contributions: one quantifying the correctness of the protocol and the other quantifying its secrecy.

The correctness term is upper-bounded by Theorem 2 of [1] (p. 16) as

(18) $$\mathrm{Pr}{\left[K_{A}\neq K_{B}\wedge F^{\mathrm{pe}}=F^{\mathrm{ec}}=✓\right]}_{\omega }\leq \varepsilon_{\mathrm{ec}}=\frac{1}{|H_{\mathrm{ec}}|}=2^{-t},$$

where *t* denotes the length of the verification hash used during error correction.

For secrecy, the problem is reduced to bounding the simplified trace-distance expression

(19) $$\left|\right|\omega_{K_{A}SCFE\wedge F=\left(✓,✓\right)}-\chi_{K_{A}}⊗\omega_{SCFE\wedge F=(✓,✓)}{\left|\right|}_{\mathrm{tr}}.$$

The security analysis introduces a scalar parameter, ν, that accounts for the unlikely event that parameter estimation passes, based on the observed error rate between registers *V* and *W*, while the fraction of mismatches between *X* and *Y* still exceeds δ by at least ν. The parameter ν thus acts as a smoothing parameter, permitting optimization over nearby quantum states in non-asymptotic entropy calculations.

Before turning to the OTP-modified protocol, we recall the final result from Theorem 3 of [1] (p. 16), which provides the secrecy bound:

(20) $$\left(\cdot \right)\leq \underset{\nu \in (0,\frac{1}{2}-\delta )}{\inf}\left\{\varepsilon_{\mathrm{pe}}\left(\nu \right)+\varepsilon_{\mathrm{pa}}\left(\nu \right)\right\},$$

with

(21) $$\varepsilon_{\mathrm{pe}}\left(\nu \right)=2\exp\left(-\frac{\left(m-k\right)k^{2}\nu^{2}}{m(k+1)}\right),$$

and

(22) $$\varepsilon_{\mathrm{pa}}\left(\nu \right)=\frac{1}{2}\sqrt{2^{-\left(m-k\right)\left(\log_{2}\frac{1}{\bar{c}}-h\left(\delta +\nu \right)\right)+r+t+l}}.$$

In addition to the quantities already introduced, the complementarity of Alice’s measurements in different bases is required in the last equation, with $\bar{c}$ defined as in [1] (p. 9). Ideally, $\bar{c}=\frac{1}{2}$. Combining Lemma 1, Theorems 2 and 3, the security of the original QKD protocol, expressed by the diamond distance, is bounded as

(23) $$\Delta \leq \varepsilon_{\mathrm{ec}}+\varepsilon_{\mathrm{pe}}\left(\nu \right)+\varepsilon_{\mathrm{pa}}\left(\nu \right).$$

The modifications to the proof for the OTP-extended protocol begin with a reformulated conclusion of Corollary 5 [1] (p. 18). In particular, the registers used for key distillation are split into a key component and an OTP component, X→XO and $Y\to Y\hat{O}$. This change yields the following uncertainty relation:

(24) $$H_{\min}^{\varepsilon }{\left(XO\wedge F^{\mathrm{pe}}=✓|VWSE\right)}_{\sigma }+H_{\max}^{\varepsilon }{\left(XO\wedge F^{\mathrm{pe}}=✓|Y\hat{O}\right)}_{\sigma }\geq \left(m-k\right)\log_{2}\frac{1}{\bar{c}},$$

where $S=S^{\Pi }S^{\Xi }S^{\Theta }$. For consistency with [1], we continue to use the designations *X* and *Y* for the raw keys prior to distillation, although the introduction of *O* and $\hat{O}$ shortens them relative to the original. This uncertainty relation bounds Eve’s maximum probability of correctly guessing Alice’s key, given her quantum side information.

To adequately account for finite-size effects and the possibility of early termination, smooth min- and max-entropies are employed. For a sub-normalized state $\rho_{AB}$, these are defined as

(25) $$H_{\min}^{\varepsilon }{\left(A|B\right)}_{\rho }:=\underset{\begin{array}{c} {\tilde{\rho }}_{AB}\in S_{\leq }\left(AB\right)\\ P({\tilde{\rho }}_{AB},\rho_{AB})\leq \varepsilon \end{array}}{\sup}H_{\min}{\left(A|B\right)}_{\tilde{\rho }}$$

and

(26) $$H_{\max}^{\varepsilon }{\left(A|B\right)}_{\rho }:=\underset{\begin{array}{c} {\tilde{\rho }}_{AB}\in S_{\leq }\left(AB\right)\\ P({\tilde{\rho }}_{AB},\rho_{AB})\leq \varepsilon \end{array}}{\inf}H_{\max}{\left(A|B\right)}_{\tilde{\rho }},$$

where $S_{\leq }\left(AB\right)$ denotes the set of sub-normalized states on AB, and P(·,·) denotes the purified distance [1] (p. 4). The smoothing parameter ε defines an ε-ball of nearby sub-normalized states around $\rho_{AB}$, which ensures the robustness of the entropy bounds against statistical fluctuations. The standard definition of the (non-smooth) conditional quantum min-entropy [1] (p. 6) is used in (25), while the conditional max-entropy in (26) follows from the duality relation $H_{\max}{\left(A|B\right)}_{\rho }:=-H_{\min}{\left(A|C\right)}_{\rho }$ for any tripartite pure state $\rho_{ABC}$.

We now apply the same modification introduced in the uncertainty relation to the bound on the conditional smooth max-entropy of the protocol state after successful parameter estimation. The adapted Proposition 8 of [1] (p. 19) gives

(27) $$H_{\max}^{\varepsilon (\nu )}{\left(XO\wedge F^{\mathrm{pe}}=✓|Y\hat{O}\right)}_{\sigma }\leq \left(m-k\right)h\left(\delta +\nu \right),$$

valid for any $\nu \in \left(0,\frac{1}{2}-\delta \right)$ such that $\varepsilon {\left(\nu \right)}^{2}<\mathrm{Pr}{\left[F^{\mathrm{pe}}=✓\right]}_{\sigma }$ and $\varepsilon \left(\nu \right)=e^{-\frac{\left(m-k\right)k^{2}\nu^{2}}{m(k+1)}}$. No additional proofs are required here, since the adjustments are purely notational, arising from the introduction of the registers *O* and $\hat{O}$.

Following the logic of Proposition 11 in [1] (p. 21), we combine the above result with the uncertainty relation to obtain

(28) $$H_{\min}^{\varepsilon (\nu )}{\left(XO\wedge F^{\mathrm{pe}}=✓|VWSE\right)}_{\sigma }\geq \left(m-k\right)q,$$

where $q=\log_{2}\frac{1}{\bar{c}}-h\left(\delta +\nu \right)$.

Discarding *W* and rewriting *V* as $C^{V}$ can be accounted for by the data-processing inequality [1] (p. 7), i.e., $H_{\min}^{\varepsilon }{\left(X|B\right)}_{\rho }\leq H_{\min}^{\varepsilon }{\left(X|C\right)}_{E(\rho )}$, valid for any CPTP map $E_{B\to C}$, yielding

(29) $$H_{\min}^{\varepsilon (\nu )}{\left(XO\wedge F^{\mathrm{pe}}=✓|SC^{V}E\right)}_{\sigma }\geq \left(m-k\right)q.$$

The OTP-encrypted error-correction data $C^{Z}$ transmitted from Alice to Bob can be integrated using the chain rule [1] (p. 7), i.e., $H_{\min}^{\varepsilon }{\left(A|BX\right)}_{\rho }\geq H_{\min}^{\varepsilon }{\left(A|B\right)}_{\rho }-\log_{2}\left|X\right|$, for a classical register *X*. Applying this to the OTP-protected exchange, similar to what is implemented for the syndrome exchange of the original protocol, we obtain

(30) $$H_{\min}^{\varepsilon (\nu )}{\left(XO\wedge F^{\mathrm{pe}}=✓|SC^{V}C^{Z}E\right)}_{\sigma }\geq \left(m-k\right)q-r_{\mathrm{noisy}},$$

where $\log_{2}\left|C^{Z}\right|$ corresponds to the OTP-specific redundancy length $r_{\mathrm{noisy}}$.

Next, we eliminate the explicit dependency on *O* in (30) by exploiting the properties of bitwise modulo-2 addition. Let us first assume a sub-normalized classical–quantum state $\rho_{XYZA}\in S_{\leq }\left(XYZA\right)$, where *X*, *Y*, and *Z* are classical registers and *A* is a quantum system possibly correlated with them. Suppose the registers are related by

(31) Z=f(X)⊕Y.

Given *X* and *Z*, the value of *Y* is uniquely determined if f(·) is known. Therefore, the non-smoothed conditional min-entropy $H_{\min}{\left(XY|ZA\right)}_{\rho }$ is equal to $H_{\min}{\left(X|ZA\right)}_{\rho }$. This can be shown first by noting that the min-entropy in the case of a classical *X* conditioned on a quantum system *B* can be expressed more conveniently using guessing probability as $H_{\min}{\left(X|B\right)}_{\rho }:=-\log_{2}p_{\mathrm{guess}}{\left(X|B\right)}_{\rho }$ [1] (p. 6). Since the classical *Y* is uniquely determined by f(X) and *Z*, if one can guess *X* correctly, one automatically knows *Y*, i.e., $p_{\mathrm{guess}}{\left(X|ZA\right)}_{\rho }=p_{\mathrm{guess}}{\left(XY|ZA\right)}_{\rho }$, where *B* is treated as ZA.

The following equalities then hold by construction:

(32) $$\begin{array}{cc} H_{\min}^{\varepsilon }{\left(XY|ZA\right)}_{\rho } & =\underset{\begin{array}{c} \tilde{\rho }\in B^{\varepsilon }\left(\rho_{XYZA}\right) \end{array}}{\sup}H_{\min}{\left(XY|ZA\right)}_{\tilde{\rho }}\\ & =\underset{\begin{array}{c} \tilde{\rho }\in B^{\varepsilon }\left(\rho_{XYZA}\right) \end{array}}{\sup}H_{\min}{\left(X|ZA\right)}_{\tilde{\rho }}=H_{\min}^{\varepsilon }{\left(X|ZA\right)}_{\rho } \end{array},$$

where the first equality follows from the definition (25), the second from the fact that non-smoothed min-entropies are maximized over the same ε-ball, and the last again from the definition. In the above, the relation between *X*, *Y*, and *Z* is enforced by construction. We smooth first before tracing out *Y*, which ensures a tight, operationally meaningful bound. On the other hand, restricting the smoothing region too early can result in a smaller ε-ball, which would lead to a less strict relation $H_{\min}^{\varepsilon }{\left(X|ZA\right)}_{\rho }\leq H_{\min}^{\varepsilon }{\left(XY|ZA\right)}_{\rho }$, which holds in general [10] (p. 82). We substitute f(·)=parity(·), Y=O, $Z=C^{Z}$, and $A=SC^{V}E$, apply the sub-normalization $F^{\mathrm{pe}}=✓$, and replace ε with ε(ν) to get

(33) $$H_{\min}^{\varepsilon (\nu )}{\left(X\wedge F^{\mathrm{pe}}=✓|SC^{V}C^{Z}E\right)}_{\sigma }=H_{\min}^{\varepsilon (\nu )}{\left(XO\wedge F^{\mathrm{pe}}=✓|SC^{V}C^{Z}E\right)}_{\sigma }.$$

With the lower bound of min-entropy established in the same form as [1],

(34) $$H_{\min}^{\varepsilon (\nu )}{\left(X\wedge F^{\mathrm{pe}}=✓|SC^{V}C^{Z}E\right)}_{\sigma }\geq \left(m-k\right)q-r_{\mathrm{noisy}},$$

we can proceed analogously. In particular, by (i) adding the independent seed $S^{H_{\mathrm{ec}}}$ for the error-correction verification hash to the left-hand side, (ii) subtracting the verification hash length *t* on the right-hand side, (iii) imposing the condition $F^{\mathrm{ec}}$ via Lemma 10 [1] (p. 21), and (iv) finalizing with Corollary 12 [1] (p. 22), we recover the results of Theorem 3 [1] (20)–(22), with the only difference being that *r* is replaced by $r_{\mathrm{noisy}}$.

Comparing the OTP-protected scheme with the original formulation for a given *m*, we first notice that the raw key length available for distillation differs. In the original scheme, *n* is equal to m−k, while in the OTP scheme, *n* is reduced to $m-k-r_{\mathrm{noisy}}$. Substituting the latter into the expression for redundancy (3), we obtain

(35) $$r_{\mathrm{noisy}}=\frac{f_{e}nh\left(\delta \right)}{1-f_{e}h\left(\delta \right)}=\frac{f_{e}\left(m-k-r_{\mathrm{noisy}}\right)h\left(\delta \right)}{1-f_{e}h\left(\delta \right)}.$$

Rearranging yields

(36) $$r_{\mathrm{noisy}}=f_{e}\left(m-k\right)h\left(\delta \right)=r.$$

In other words, although $r_{\mathrm{noisy}}$ exceeds *r* for correcting the same message length, in the OTP-protected variant, the effective message length is shortened. This ensures that, for a given *m* and Δ, the redundancy parameters $r_{\mathrm{noisy}}$ and *r* match exactly.

Consequently, the achievable key rate l/m is identical in both schemes for a fixed security parameter Δ. In this respect, the OTP-extended and original protocols are therefore equivalent. Since (32) is exact, while the rest of the proof reuses the bounds of the original proof, the Δ bound is as strict as in the reference scheme.

## 7. Error-Correction Performance

We demonstrate the benefits of the OTP scheme using LDPC codes, which achieve performance close to the Shannon limit for reliable communication over noisy channels. First introduced by Gallager in 1962 [11], LDPC codes became practical with the rise of efficient computational techniques and are now widely deployed. Their main feature is a sparse parity-check matrix—predominantly zeros with relatively few ones—enabling efficient iterative decoding, most commonly implemented via belief-propagation or message-passing algorithms.

In the following, we compare syndrome-based error correction with the parity-based OTP approach. The notation used to describe the error-correction algorithms is summarized in Table 2.

In the syndrome-based method, Alice holds a sparse parity-check matrix *H* of size r×(m−k). She calculates the syndrome of her raw key as

(37) $$s_{A}=Hk_{A},$$

where $k_{A}$ denotes her raw key represented as a column vector of length n=m−k, and all operations are performed modulo 2. The rows of *H* correspond to *r* parity-check equations.

After obtaining $s_{A}$, Bob applies an LDPC decoding algorithm to recover the most probable candidate $k_{B}^{\prime }$, i.e., the vector closest to his raw key $k_{B}$, subject to the condition

(38) $$H_{\mathrm{ext}}\left[\begin{array}{c} k_{B}^{\prime }\\ s_{A} \end{array}\right]=0,$$

where $H_{\mathrm{ext}}=\left[H|I\right]$ denotes the identity-extended parity-check matrix.

Equivalently, the process can be described in terms of error vectors. Bob first computes his own syndrome $s_{B}=Hk_{B}$, then identifies the most likely error vector e satisfying $s_{A}=s_{B}+He$, and finally reconstructs Alice’s key $k_{B}^{\prime }=k_{B}+e$. Both formulations are equivalent and lead to the same corrected key.

In contrast, parity-based error correction over the noisy channel requires Alice to employ a parity-generator matrix *P* of size $r_{\mathrm{noisy}}\times \left(m-k-r_{\mathrm{noisy}}\right)$. This matrix is derived from the decomposition $H=\left[A|B\right]$, where *A* is a submatrix of size $r_{\mathrm{noisy}}\times \left(m-k-r_{\mathrm{noisy}}\right)$, corresponding to parity-check equations for the message bits, and *B* is a square submatrix of dimension $r_{\mathrm{noisy}}\times r_{\mathrm{noisy}}$ corresponding to parity-check equations for the parity bits themselves. The generator matrix can be calculated as

(39) $$P=B^{-1}A.$$

Alice first computes her parity bits as

(40) $$p_{A}=Pk_{A},$$

where $k_{A}$ is represented as a column vector of length $n=m-k-r_{\mathrm{noisy}}$. The OTP encryption $p_{\mathrm{enc}}=p_{A}+o_{A}$ followed by the decryption $p_{B}=p_{\mathrm{enc}}+o_{B}$ on Bob’s side effectively emulates the transmission of parity bits through a noisy channel, where $o_{A}$ and $o_{B}$ are one-time pad vectors on the respective sides.

After obtaining $p_{B}$, Bob employs LDPC decoding algorithms to find the most probable key $k_{B}^{\prime }$ that is closest to $k_{B}$ by solving the equation

(41) $$H\left[\begin{array}{c} k_{B}^{\prime }\\ p_{B} \end{array}\right]=0.$$

Although $H_{\mathrm{ext}}$ is larger than *H*, the decoding task (38) can be considered just as difficult as (41). This is because, in the syndrome-based approach, the received syndrome $s_{A}$ in (38) is already correct, while in the OTP scheme, the errors are present in $k_{B}^{\prime }$ and $p_{B}$. The combined length of these erroneous components is equal to the length of $k_{B}^{\prime }$ alone in the syndrome case, under the condition $r_{\mathrm{noisy}}=r$. The advantage of the OTP scheme is on Alice’s side: because of the smaller matrix dimensions, computing (40) requires about $r^{2}$ fewer operations than computing (37). This estimate is approximate, since different algorithmic optimizations may be applied in practice.

The decoding tasks are comparable, and thus the robustness of the QKD protocol—measured by the success probability Pr[F=(✓,✓)]—remains essentially the same for both schemes, subject to some random variations. In contrast, the encoding tasks differ in difficulty: when expressed as the ratio of encoding problem sizes, determined by the number of elements in *P* and *H* and using (36), we obtain

(42) $$\frac{r\times (m-k-r)}{r\times (m-k)}=1-\frac{r}{m-k}=1-\frac{f_{e}\left(m-k\right)h\left(\delta \right)}{m-k}=1-f_{e}h\left(\delta \right).$$

Since the same parity-check matrix can be reused across multiple sessions, recalculating *P* for each session is unnecessary. To compute (39), the submatrix *B* must be nonsingular; however, the explicit inversion of *B* is not required to generate the parity bits. Instead, an LU decomposition of *B* can be performed, where B=LU, with *L* a lower triangular matrix and *U* an upper triangular matrix. By heuristically rearranging the rows and columns of *H*, both *L* and *U* can be made sparse. This enables efficient calculation of the parity bits using standard forward and backward substitution.

In practice, the raw key is segmented to allow the use of parity-check matrices of manageable size, suitable for software implementation or, preferably, for efficient hardware implementation. For example, 5G parity-check matrices [12] (pp. 19–26) can be employed. Using the 5G Base Graph 1 matrix with a lifting factor of 224, a matrix *H* of size 5072 × 10,000 can be extracted, which experimentally achieves an error-correction success rate of 0.99 at a bit error rate of 0.09. The resulting error-correction efficiency is $f_{e}\approx 1.16$, since the theoretical redundancy is 10,000h(0.09)=4365. The corresponding parity-generator matrix *P* then has the dimensions 5072×4928, giving a ratio of (42) equal to 0.4928. In other words, under this setup, syndrome encoding is approximately twice as demanding as parity encoding when assessed purely in terms of problem size.

In the syndrome scheme, *H* is employed to generate error-correction data, whereas in the OTP scheme it is primarily used to verify parity-check equations on the receiver side, an approach more in line with classical wireless communication. One could alternatively define *H* as the parity generator in the OTP case, but this would break the equivalence $r_{\mathrm{noisy}}=r$, since the problem dimensions would no longer align.

The final key length *l* and the parameters *k*, *t*, and ν for a given Δ, *m*, δ, and $\bar{c}$ are identical across both schemes. However, since the length of the distilled keys *n* is different, the compression ratio used in privacy amplification must be adjusted accordingly. In particular, the OTP scheme requires a compression ratio that is only a fraction of the ratio of the reference scheme,

(43) $$\frac{n_{\mathrm{otp}}}{l}/\frac{n_{\mathrm{ref}}}{l}=\frac{m-k-r}{m-k}=1-\frac{f_{e}\left(m-k\right)h\left(\delta \right)}{m-k}=1-f_{e}h\left(\delta \right),$$

which is independent of *m* and coincides with the size ratio of the encoding problems (42).

**Figure 2:**
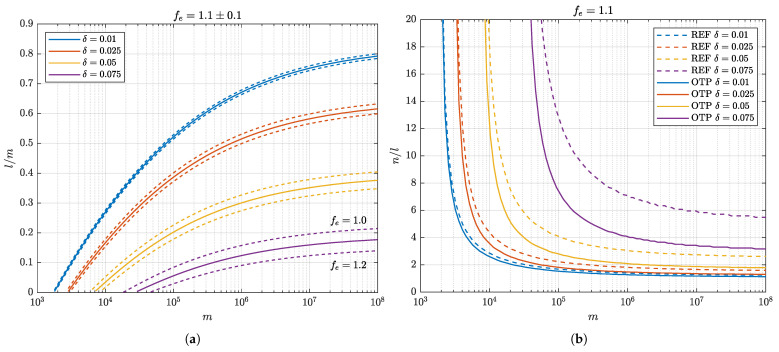
(**a**) Achievable key rates l/m for the reference syndrome-based scheme and the OTP-protected scheme, computed at security parameter $\Delta \leq 10^{-10}$ and error-correction efficiency $f_{e}=1.1\pm 0.1$. Both schemes achieve identical key rates. (**b**) Required compression ratios in the privacy amplification step for the reference syndrome-based scheme and the OTP-protected scheme, under the same parameters as in (**a**) and $f_{e}=1.1$. The OTP scheme requires less compression due to shorter distilled key lengths, reflecting the reduction in effective problem size.

Figure 2a presents the achievable key rates l/m determined by numerical optimization for $\Delta \leq 10^{-10}$ and error-correction efficiency $f_{e}=1.1$ within a range of ±0.1. Figure 2b shows the corresponding compression ratios under $f_{e}=1.1$.

## 8. Conclusions

The use of one-time pad protection in QKD has been proposed before, often accompanied by the claim that the encryption of the error-correction syndrome ensures that any remaining information leakage becomes useless to an eavesdropper. Such assertions, however, are generally only valid under restrictive assumptions and specific settings. In particular, prior work often leaves unaddressed the generation and potential leakage of pre-shared encryption keys, or relies on session chaining without adequately analyzing the security of the initial conditions. Moreover, the information available to an adversary is not limited to the public communication channel; consequently, the analysis is incomplete.

In this work, we show that the information disclosed over the public channel is determined by the choice of error-correction scheme, regardless of whether the data is encrypted, if the generation of encryption keys is also part of a security framework. Here, the overall security is evaluated in terms of the protocol’s distinguishability from the ideal QKD protocol. While the exact computation of the diamond distance between the corresponding CPTP maps is challenging, we can reuse much of the non-asymptotic treatment developed by Tomamichel and Leverrier. Apart from this theoretical equivalence, the OTP approach also offers practical implementation advantages. As shown for LDPC codes, it has the potential to reduce the computational resources required for error correction. As QKD is increasingly integrated into related technologies such as quantum secure direct communication [13], new opportunities for future research arise, in terms of both theoretical security and performance aspects of error correction.

## Figures and Tables

**Table 1:** Notation used in the modeling and proof.

Δ	Diamond distance between two CPTP maps
*A*, *B*	Tensor product of Alice’s and Bob’s individual quantum systems
*E*	Purification representing Eve’s quantum memory
*m*	Number of shared quantum systems measured in the same bases (raw key)
*k*	Number of bits of the raw key for parameter estimation
*n*	Number of bits of the raw key for key distillation
*l*	Length of the final key
*t*	Length of the hash for the error-correction check
*r*, $r_{\mathrm{noisy}}$	Redundancy length in the syndrome and OTP scheme
$f_{e}$	Error-correction (in)efficiency factor
δ	Threshold value for parameter estimation
ν	Smoothing parameter indicating the increase in differences over δ in the non-parameter estimation part of the raw key
$\bar{c}$	Complementarity of Alice’s measurements
$H_{\mathrm{ec}}$	Family of universal hash functions used to check the success of error correction
$H_{\mathrm{pa}}$	Family of universal hash functions used for privacy amplification
$S^{\Pi }$	Seed for the choice of a subset of raw key bits for parameter estimation
$S^{\Xi }$	Seed for the choice of measurement bases for parameter estimation
$S^{\Theta }$	Seed for the choice of measurement bases for key distillation
$S^{H_{\mathrm{ec}}}$	Seed for the selection of the hash function for checking the error correction
$S^{H_{\mathrm{pa}}}$	Seed for the selection of the hash function for privacy amplification
$F^{\mathrm{pe}}$	Flag indicating the failure of the parameter estimation
$F^{\mathrm{ec}}$	Flag indicating the failure of the error correction
*V*, *W*	Alice’s and Bob’s registers with classical bits for parameter estimation
*X*, *Y*	Alice’s and Bob’s registers with classical bits for key distillation
*O*, $\hat{O}$	Alice’s and Bob’s registers with classical bits of the OTP key
$\hat{X}$	Register with Bob’s version of the key *X*
*Z*	Register with the OTP-encrypted error-correction data
*P*	Register containing Alice’s error-correction data
$\hat{P}$	Register containing Bob’s error-correction data with noise
*T*, $\hat{T}$	Alice’s and Bob’s registers with distilled key hash
$C^{V}$	Transcript of the register *V* disclosed on the public channel
$C^{Z}$	Transcript of the register *Z* disclosed on the public channel
$C^{T}$	Transcript of the register *T* disclosed on the public channel
$K_{A}$, $K_{B}$	Registers containing Alice’s and Bob’s final keys
χ	Quantum representation of an ideal key
ρ	Quantum state before any measurement
τ	Quantum state after parameter estimation
σ	Quantum state after error correction
ω	Final quantum state

**Table 2:** Notation used in describing error-correction algorithms.

*H*	Parity-check matrix of size r×(m−k)
*A*	Submatrix of $H=\left[A|B\right]$ of size $r_{\mathrm{noisy}}\times \left(m-k-r_{\mathrm{noisy}}\right)$
*B*	Square submatrix of $H=\left[A|B\right]$ of size $r_{\mathrm{noisy}}\times r_{\mathrm{noisy}}$
*P*	Parity-generator matrix of size $r_{\mathrm{noisy}}\times \left(m-k-r_{\mathrm{noisy}}\right)$
$H_{\mathrm{ext}}$	Identity-extended parity-check matrix of size r×(m−k+r)
*L*, *U*	Lower and upper triangular decomposition of *B*
$k_{A}$, $k_{B}$	Alice’s and Bob’s raw keys for distillation of length m−k (syndrome scheme) and $m-k-r_{\mathrm{noisy}}$ (parity scheme)
$s_{A}$, $s_{B}$	Alice’s and Bob’s key syndromes of length *r*
$o_{A}$, $o_{B}$	Alice’s and Bob’s one-time pad vectors of length $r_{\mathrm{noisy}}$
$p_{A}$	Parity data of Alice’s key of length $r_{\mathrm{noisy}}$
$p_{B}$	Parity data of Alice’s key with noise on Bob’s side of length $r_{\mathrm{noisy}}$
$p_{\mathrm{enc}}$	OTP-encrypted parity data of length $r_{\mathrm{noisy}}$
e	Bob’s most likely error vector of length m−k
$k_{B}^{\prime }$	Bob’s most likely key of length m−k (syndrome scheme) and $m-k-r_{\mathrm{noisy}}$ (parity scheme)

## Data Availability

No new data were created or analyzed in this study.

## Abbreviations

The following abbreviations are used in this manuscript:

BSC	Binary symmetric channel
CPTP	Completely positive trace-preserving
FEC	Forward error correction
LDPC	Low-density parity-check
OTP	One-time pad
QKD	Quantum key distribution
